# Herb-Drug Interaction Between Xiyanping Injection and Lopinavir/Ritonavir, Two Agents Used in COVID-19 Pharmacotherapy

**DOI:** 10.3389/fphar.2021.773126

**Published:** 2021-11-11

**Authors:** Linhu Ye, Lei Cheng, Yan Deng, Hong Liu, Xinyu Wu, Tingting Wang, Qi Chang, Yan Zhang, Dan Wang, Zongze Li, Xixiao Yang

**Affiliations:** ^1^ Department of Pharmacy, Shenzhen Hospital, Southern Medical University, Shenzhen, China; ^2^ Department of Pharmacy, Bijie City First People’s Hospital, Bijie, China; ^3^ Institute of Medicinal Plant Development, Chinese Academy of Medical Sciences, Peking Union Medical College, Beijing, China

**Keywords:** coronavirus disease 2019, herb-drug interactions, xiyanping injection, lopinavir/ritonavir, CYP3A4

## Abstract

The global epidemic outbreak of the coronavirus disease 2019 (COVID-19), which exhibits high infectivity, resulted in thousands of deaths due to the lack of specific drugs. Certain traditional Chinese medicines (TCMs), such as Xiyanping injection (XYPI), have exhibited remarkable benefits against COVID-19. Although TCM combined with Western medicine is considered an effective approach for the treatment of COVID-19, the combination may result in potential herb-drug interactions in the clinical setting. The present study aims to verify the effect of XYPI on the oral pharmacokinetics of lopinavir (LPV)/ritonavir (RTV) using an *in vivo* rat model and *in vitro* incubation model of human liver microsomes. After being pretreated with an intravenous dose of XYPI (52.5 mg/kg) for one day and for seven consecutive days, the rats received an oral dose of LPV/RTV (42:10.5 mg/kg). Except for the t_1/2_ of LPV is significantly prolonged from 4.66 to 7.18 h (*p* < 0.05) after seven consecutive days pretreatment, the pretreatment resulted in only a slight change in the other pharmacokinetic parameters of LPV. However, the pharmacokinetic parameters of RTV were significantly changed after pretreatment with XYPI, particularly in treatment for seven consecutive days, the AUC_0-∞_ of RTV was significantly shifted from 0.69 to 2.72 h μg/mL (*p* < 0.05) and the CL exhibited a tendency to decrease from 2.71 L/h to 0.94 L/h (*p* < 0.05), and the t_1/2_ of RTV prolonged from 3.70 to 5.51 h (*p* < 0.05), in comparison with the corresponding parameters in untreated rats. After administration of XYPI, the expression of Cyp3a1 protein was no significant changed in rats. The *in vitro* incubation study showed XYPI noncompetitively inhibited human CYP3A4 with an apparent *Ki* value of 0.54 mg/ml in a time-dependent manner. Our study demonstrated that XYPI affects the pharmacokinetics of LPV/RTV by inhibiting CYP3A4 activity. On the basis of this data, patients and clinicians can take precautions to avoid potential drug-interaction risks in COVID-19 treatment.

## Introduction

The global outbreak of coronavirus disease 2019 (COVID-19), which exhibits high infectivity, resulted in thousands of deaths; it is considered a major global public health emergency presenting several negative effects and unprecedented challenges (Gates, 2020). Identifying effective medications and defining optimal therapies for COVID-19 patients are urgent tasks with huge challenges, particularly for patients with severe pneumonia or/and complex systemic manifestations. Due to the long life cycle and difficulty in developing new drugs, certain approved for marketing medications including chemical and herbal medications have been used for COVID-19 therapy in clinical trials. In China, except for chemicals, such as lopinavir/ritonavir (LPV/RTV) (Cao et al., 2020), arbidol (Wang et al., 2020) and chloroquine (Gao et al., 2020) have shown efficacy in COVID-19 patients. Traditional Chinese medicines (TCMs) also have exhibited remarkable benefits against COVID-19 in the clinical trials (Gao, 2020), especially certain traditional Chinese patent medicines such as Lianhua Qingwen capsule, Qingfei Paidu decoction, and Xuebiqing injection (Zhuang et al., 2020; Zhao et al., 2021).

Xiyanping injection (XYPI) is prepared from a well-known TCM, named *Andrographis paniculata* (Burm. f.) Nees; the major active components of the injection are sulfonated andrographolides including andrographolide sulfate A, andrographolide sulfate B, andrographolide sulfate C, and so on (Zhan et al., 2012). Modern pharmacological studies indicate that it has antiviral, antipyretic, and anti-inflammatory effects (Zhuang et al., 2020). It is used for the treatment of upper respiratory tract infections, viral pneumonia, bronchitis, and bacillary dysentery in the clinic. Additionally, it is recommended for the treatment of human infection with H7N9 avian influenza, according to China’s Diagnosis and Treatment Scheme for Human Infection with H7N9 Avian Influenza (Version 2017). Recently, XYPI was reported to be effective in the treatment of COVID-19 (Cai N. et al., 2020; Guo et al., 2020), and it was recommended as a treatment agent in the Guideline on Diagnosis and Treatment of COVID-19 by the National Health Commission of the People’s Republic of China and National Administration of Traditional Chinese Medicine (Trial eighth Edition). It was used for the critical syndrome of exuberance of internal heat toxin in the progressive stage of COVID-19 such as fever, sore throat, cough with yellow phlegm, and chest distress (Zhuang et al., 2020). Combination of drugs is considered an effective approach for the prevention and treatment of COVID-19 (Qu et al., 2020; Wang et al., 2021). A commonly used combination in the treatment of COVID-19 in the clinic is XYPI with LPV/RTV (a fixed-dose combination at a 4:1 ratio) tablets (Zhuang et al., 2020).

As is known to all, pharmacokinetic and pharmacodynamic interactions are two basic types of drug interactions in clinical, and the former is the main type because it may occur at multiple stages including absorption, distribution, metabolism, and/or excretion. Most drug interactions involve in the activity of the major drug-metabolizing enzymes or/and drug transporters such as cytochrome P450 (CYP or P450) enzymes and/or P-glycoprotein (P-gp) (König et al., 2013; Zhang et al., 2021a). LPV and RTV are primarily metabolized by the P450; both are substrates of CYP3A4 and P-gp, as well as highly protein bound in the plasma. These characteristics increase the tendency of drug interactions when they are combined with other drugs. For example, the pharmacokinetic profiles of LPV were affected by Qingfei Paidu decoction, Jingyin granules (two traditional Chinese patent medicines recommended for combating COVID-19 in China) (Zhang et al., 2021a; Zhang et al., 2021b) and Tang herb (a Chinese patent medicine approved for use in HIV positive patients) (Yao et al., 2014). Moreover, infection and inflammation are associated with down-regulation of hepatic and extra-hepatic expression and/or activities of P450, especially CYP3A4 (Morgan, 2009). In addition, our previous study demonstrated that XYPI has a strong inhibitory effect on CYP3A4 and CYP2E1 (Ye et al., 2014). Thus, the concomitant use of XYPI and LPV/RTV may trigger potential herb-drug interactions (HDIs) and lead to unexpected adverse drug reactions in clinical settings. Therefore, it is urgent and essential to assess the potential risks of HDIs in COVID-19 treatment.

The present study was performed to investigate whether XYPI can affect the oral pharmacokinetics of LPV/RTV in rats, using a sensitive and reliable LC-MS/MS method to analyze LPV and RTV. For further understanding the possible mechanisms of herb-drug interactions between XYPI and LPV/RTV, the expression of Cyp3a1 protein in rats was analysed by western blotting, and the inhibitory effect of XYPI on CYP3A4 activity was explored through incubation of testosterone with human liver microsomes.

## Material and Methods

### Chemicals and Reagents

RTV, LPV, and indinavir sulfate were purchased from Macklin Inc. (Shanghai, China); testosterone was purchased from Sigma-Aldrich (St. Louis, MO, United States); 6β-hydroxytestosterone was purchased from Toronto Research Chemicals Inc. (Toronto, Canada); β-nicotinamide adenine dinucleotide phosphate (NADPH) was purchased from Roche (Roche, Switzerland); propranolol was obtained from the National Institutes for Food and Drug Control (Beijing, China); Solutol HS-15 was purchased from MedChemExpress LLC (Shanghai, China); and XYPI was purchased from Jiangxi Qingfeng Pharmaceutical Co. Ltd. (Jiangxi, China). A pooled sample of human liver microsomes (HLM, 452161) was obtained from BD Gentest Corporation (BD Gentest TM, Woburn, MA, United States). Antibodies specific for CYP3A1 (Mouse monoclonal antibody) was purchased from Santa Cruz Biotechnology (Cruz, CA, United States). Primary antibody for GAPDH (Rabbit Polyclonal antibody) and the second-antibody specific for Rabbit and Mouse were purchased from Proteintech Group, Inc. Methanol and acetonitrile of high-performance liquid chromatography (HPLC) grade were obtained from Fisher Co. Ltd. (Waltham, MA, United States). Milli-Q (Millford, MA, United States) water was used throughout the study.

### Animals

Male Sprague-Dawley rats (200 ± 20 g) were supplied by SJA Laboratory Animal Co. Ltd. (Hunan, China) and housed under standard conditions of temperature, humidity, and light, with free access to standard rodent diet and water before the experiment. The animal experiment was approved by the Animal Ethics Committee at Bijie City First People’s Hospital. On the day before LPV and RTV administration, the rats were subjected to a light surgery. A polyethylene catheter (0.50 mm ID, 1.00 mm OD; Portex Limited, Hythe, Kent, England) was cannulated into the right jugular vein under anesthesia induced through intraperitoneal administration of 10% chloral hydrate at 3.50 ml/kg. After surgery, the rats were placed individually in metabolism cages to allow recovery for at least 24 h. The rats were fasted overnight with free access to water prior to LPV and RTV administration.

### Effect of XYPI on the Pharmacokinetics of LPV and RTV

#### Drug Administration and Blood Sample Collection

The rats were randomly divided into three groups (n = 6, for each group). They received an intravenous (*iv*) dose of 52.5 mg/kg XYPI for groups 1 (1 day) and 2 (7 consecutive days) and a vehicle (normal saline) for group 3, once per day. Two hours after the last dose of XYPI *via* the vehicle, the rats were orally administered LPV/RTV (42/10.5 mg/kg) dissolved in Solutol HS-15 (8%). Blood samples (0.4 ml) were collected in heparinized centrifuge tubes through the catheter at time points of 0.5, 1, 2, 3, 4, 5, 6, 8, 10, 12, and 24 h after dosing and centrifuged immediately at 3,000 *g*, 4°C for 8 min. The separated plasma samples were stored at −40°C until analysis. After drug administration and blood collection, 0.2 ml of normal saline containing 20 units of heparin was injected into the body *via* the catheter to flush the catheter and prevent coagulation.

#### Sample Preparation

A 100 μL aliquot of rat plasma was spiked with 10 μL of IS solution (indinavir 5.0 μg/ml) and, subsequently, vortex-mixed with 390 μL of acetonitrile for 10 min for protein precipitation. After centrifugation of the samples at 8,000 *g* for 5 min, 200 µL of the supernatant was transferred, and 10 µL of the resulting sample solution was injected into an LC-MS/MS system for analyzing LPV and RTV.

### Inhibitory Effect of XYPI on CYP3A4 Activity

The effect of XYPI on human CYP3A4 activity was assayed according to the method used in our previous study (Ye et al., 2016). Briefly, an incubation mixture containing 0.25 mg/ml liver microsomal protein, 100 mM phosphate buffer (pH 7.4), 3.3 mM MgCl_2_, and 50 µM testosterone was pre-incubated for 5 min in a shaking water bath at 37°C. The metabolism reaction was initiated by adding 1 mM NADPH. The total volume of the incubation mixture was 100 µL. After 30 min of incubation, the reaction was stopped by adding 100 µL of ice-cold acetonitrile containing propranolol (used as IS, 1 μg/ml). The mixtures was vortex-mixed and, subsequently, centrifuged at 15,000 *g* and 4°C for 5 min. A 10 µL aliquot of the supernatant was injected into an LC-MS/MS system for analyzing the metabolite 6β-hydroxytestosterone. To further understand the inhibitory model and inhibition constant *Ki* for XYPI-induced inhibition of CYP3A4 isoenzyme activity, the above incubation was performed with different concentrations of testosterone (from 10 to 100 µM) or XYPI (from 0 to 4 mg/ml) in triplicates.

Furthermore, different concentration XYPI samples were preincubated with HLM. After various preincubation times (0, 5, 10, and 20 min) in a shaking water bath at 37°C, 10 µL of the substrate and 10 µL of NADPH were added, and the reaction was continued for 30 min under the same conditions. The reaction was stopped by the addition of 100 µL of ice-cold acetonitrile containing 1 μg/ml propranolol. The incubation mixtures were then centrifuged at 15,000 g for 10 min at 4°C. Each incubation test was performed in triplicate, and 10-µL aliquots of the supernatants were injected into an LC-MS/MS system for analyzing the metabolite 6β-hydroxytestosterone.

### Analysis of 6β-Hydroxytestosteronewas

The concentrations of 6β-hydroxytestosterone was analyzed using an LC-MS/MS system consisting of an Agilent 1200 HPLC (Palo Alto, CA, United States) and an Applied Biosystem 3200 Q-Trap (Foster City, CA, United States) equipped with an electrospray ionization interface. The 10-µL aliquots of the samples were separated on a RP-C18 column (2.1 × 50 mm i. d, 3.5-µM particle size; Agilent, United States) maintained at 40°C. The mobile phase consisted of 0.1% formic acid in water (A) and in acetonitrile (B) with a flow rate of 0.4 ml/min. Phase A was linearly increased from 5 to 90% over a period of 0.1 min, maintained at 90% for an additional 4 min, and then decreased to 5% for re-equilibration; the total run time was 7 min. The TurboIonSpray interface was operated separately in the positive ion mode at 5500 V. The operating conditions were the following: ion source temperature, 400°C; curtain gas, 20 psi; ion source gas 1, 60 psi; ion source gas 2, 60 psi. The quantification was performed by multiple reaction monitoring (MRM) of the molecular ion and the related product ion. The MRM transitions (collision energy) of 6β-hydroxytestosterone, and propranolol, (IS) were m/z 305.1→269.3 (25 eV), and 261.3→116.1 (32 eV), respectively.

### Analysis of LPV and RTV

The concentrations of LPV and RTV were analyzed using an LC-MS/MS system consisting of an Agilent 1260 HPLC system (Palo Alto, CA, United States) and an Applied Biosystem 4500 Q-Trap system (Foster City, CA, United States). The chromatographic separation was performed on an RP-C_18_ column (2.1 mm × 50 mm, i. d, 3.5 µM particle size; Agilent, United States) maintained at 30°C. The mobile phase consisted of 0.1% formic acid in water (A) and acetonitrile (B); it was run according to the following gradient programs at a flow rate of 0.4 ml/min: 85% A (0–0.1 min), 85–10% A (0.1–2.1 min), and 10–85% A (2.1–4.0 min) for the LPV and RTV assays. The mass detector with electrospray ionization interface was operated in positive ion mode and set according to the following conditions: spray voltage, 5,500 V; spray temperature, 450 °C; curtain gas, 20 psi; source gas 1, 60 psi; and source gas 2, 60 psi. The quantification was performed through MRM of the molecular ion to related product ion for each compound. The MRM transitions (collision energy) of LPV, RTV, and indinavir (IS) were *m/z* 629.5→155.1 (28 eV), 721.3→268.2 (32 eV), and 614.4→421.1 (32 eV), respectively. The peak area ratio of the analyzed LPV or RTV versus IS was used for calculating the concentration.

### Western Blotting

Liver tissues were lysed with ice-cold RIPA buffer. Equal amount of proteins of the samples were loaded onto the gel. After electrophoretic separation, the proteins were transferred to a nitrocellulose membrane (0.2 μm). After incubation in blocking buffer (5% non-fat milk in PBS), the membranes were subsequently incubated with specific antibodies to perform immunoblot analyses.

### Data Analysis

The following pharmacokinetic parameters were estimated from the plasma concentration-time curve for each rat using a non-compartmental model of the WinNonlin software (Pharsight, Mountain View, CA, United States): peak plasma concentration (C_max_); time (T_max_) to C_max_; terminal elimination half-life (t_1/2_); area under the plasma concentration-time curve (AUC) from zero to the last sample collection time (AUC_0-t_); AUC from zero to infinity (AUC_0-∞_); Total body clearance (CL), volume of distribution (Vd), and mean residence time (MRT). All data are expressed as the mean ± SD. Statistical analysis was performed by analysis of variance (ANOVA). In brief, statistical analysis (group 1 = XXPI (1 day) +LPV, group 2 = XXPI (7 days) + LPV), and group 3 = LPV (Control) were performed by one way ANOVA *via* SPSS 16.0 statistic software, dependent variable included the tested pharmacokinetic parameters such as Tmax, Cmax, t_1/2, λz_ and so on. Post Hoc analysis (group 1 vs group 3, and group 2 vs group 3) are performed by LSD test (equal variances assumed) or Dunnett’s test (equal variances not assumed). And a *p* value between groups less than 0.05 was considered significant.

The mode of inhibition was initially estimated graphically from the Lineweaver-Burk plots, and the *Ki* values were ultimately determined through nonlinear regression analysis based on the best enzyme inhibition model using GraphPad Prism 5.0 (GraphPad Software Inc., United States). Each data point represents the average of triplicate measurements.

## Result

### Method Validation for LPV and RTV Determination

**FIGURE 1:**
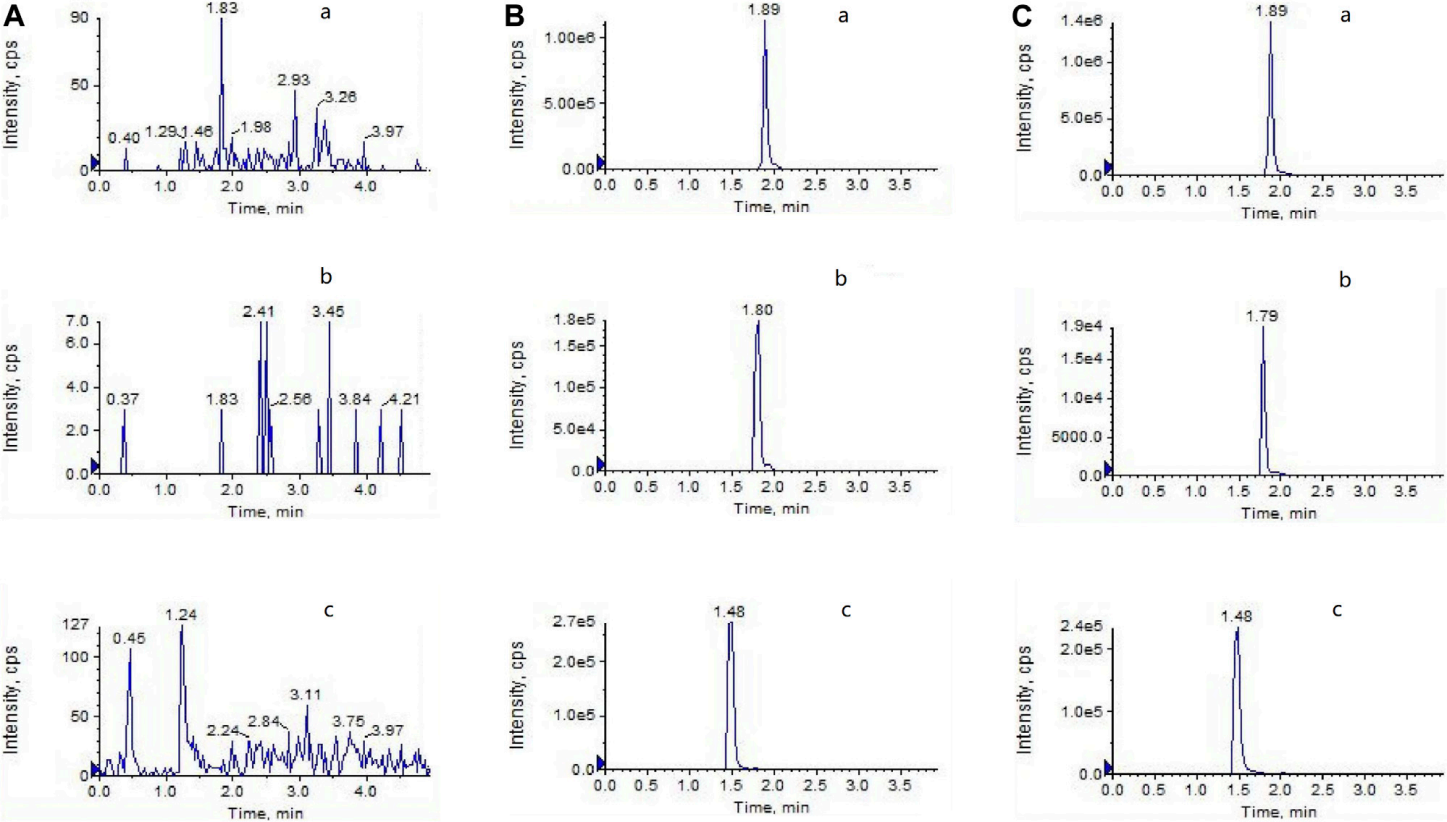
LC-MS/MS chromatograms of **(A)** blank plasma **(B)** blank plasma spiked with LPV (3,000 ng/ml), RTV (300 ng/ml), and indinavir as the IS (100 ng/ml) **(C)** plasma at 3 h after oral administration of LPV/RTV (42:10.5 mg/kg).

Before the pharmacokinetic study, the LC-MS/MS method was validated. Figure 1 depicts representative LC-MS/MS chromatograms of the blank plasma (Figure 1A), quality control plasma sample (blank plasma supplemented with LPV 3,000 ng/ml), RTV (300 ng/ml), and IS (indinavir 100 ng/ml), respectively; Figure 1B), and plasma samples collected 3 h after oral administration of LPV/RTV (Figure 1C). The chromatograms reveal that LPV, RTV, and IS were completely separated using HPLC with retention times of 1.89, 1.80, and 1.48 min, respectively. The calibration curves between the peak area ratios of LPV/IS and RTV/IS against LPV and RTV concentrations of 30–9,000 ng/ml and 5–2,000 ng/ml, respectively, showed good linearity, with all correlation coefficients higher than 0.99. The LLOD and LLOQ were 5 ng/ml and 30 ng/ml, respectively, for LPV, and 2 ng/ml and 5 ng/ml, respectively, for RTV. The method showed good reproducibility with intra-day (n = 6) and inter-day (n = 6, 3 days) precision less than 10.66 and 7.20%, respectively, and excellent accuracy ranging from 94.16 to 110.63% and 91.67–102.68% for LPV and RTV, respectively (Supplementary Table S1). The mean extraction recoveries were between 97.06 to 115.92% and 93.92–101.55%, respectively, for LPV and RTV. The accuracy of dilution for 18 μg/ml LPV (n = 6) was 106.83%. These values meet the United States Food and Drug Administration criteria for the validation of bio-analytical methods.

### Effect of XYPI on the Pharmacokinetics of LPV and RTV in Rats

**FIGURE 2:**
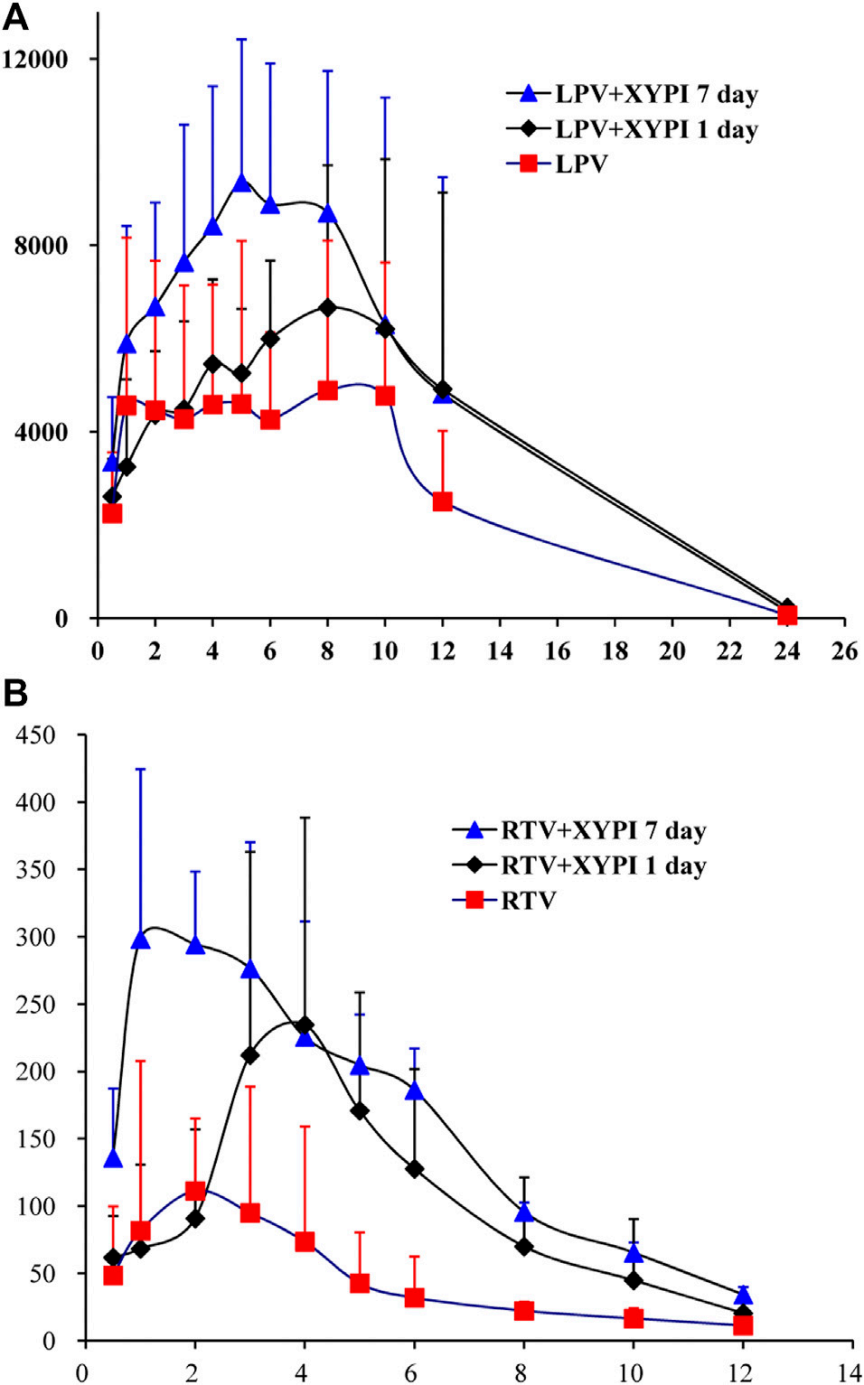
Mean plasma concentration-time profiles of LPV **(A)** and RTV **(B)** following oral administration of LPV/RTV (42:10.5 mg/kg) in rats that were pretreated with or without XYPI (x±SD, n = 6).

After being pretreated with an intravenous dose of XYPI (52.5 mg/kg) for 1 day or for seven consecutive days, the rats received an oral dose of LPV/RTV. The plasma concentration versus time profiles of LPV and RTV are shown in Figure 2A and Figure 2B, and relevant pharmacokinetic parameters are presented in Tables 1, 2. These data reveal that certain parameters of LPV and RTV were significantly changed in the rats after XYPI treatment for seven consecutive days. The pharmacokinetic parameters of LPV was only slightly changed in the groups subjected to 1 day and seven consecutive days of XYPI administration compared to that in the control group. Except for the t_1/2_ of LPV is significantly prolonged from 4.66 to 7.18 h [F (3, 15) = 3.237, *p* < 0.05] after seven consecutive days pretreatment. However, the half-life of RTV was significantly prolonged from 3.70 to 6.55 h and 5.51 h [F (3, 15) = 4.900, *p* < 0.05] after treatment for one day and seven consecutive days, respectively. The AUC_0-∞_ of RTV was significantly shifted from 0.69 to 2.72 h μg/mL [F (3, 15) = 6.815, *p* < 0.05] and CL was significantly exhibited a tendency to decrease from 2.71 L/h to 0.94 L/h [F (3, 15) = 6.199, *p* < 0.05] after treatment for seven consecutive days. These results revealed that continuous administration of XYPI can change the metabolism of LPV and RTV.

**TABLE 1:** Pharmacokinetic parameters of LPV after oral administration of LPV/RTV (42:10.5 mg/kg) in rats that were pretreated with or without XYPI ( x(_) ±SD, n = 6).

Parameters	LPV (control)	XXPI (1 day) +LPV	XXPI (7 days) + LPV
Tmax (h)	6.17 ± 4.22	7.16 ± 3.60	6.25 ± 2.28
Cmax (μg/ml)	9.94 ± 1.83	8.63 ± 3.13	11.88 ± 2.77
t_1/2_, _λz_ (h)	4.66 ± 0.98	5.89 ± 2.78	7.18 ± 2.71*
AUC_0-24_ (μg·h/mL)	86.11 ± 18.42	109.46 ± 46.25	131.32 ± 47.26
AUC_0-∞_ (μg·h/mL)	86.60 ± 18.24	111.89 ± 46.01	133.02 ± 47.63
V_d_ (L)	34.31 ± 9.63	36.55 ± 25.17	38.26 ± 23.41
CL (L/h)	5.14 ± 1.12	4.24 ± 1.39	3.58 ± 1.11
MRTINF (h)	7.90 ± 1.67	9.08 ± 1.00	7.80 ± 0.98

*p< 0.05, significantly different compared with control group.

**TABLE 2:** Pharmacokinetic parameters of RTV after oral administration of LPV/RTV (42:10.5 mg/kg) in rats that were pretreated with or without XYPI ( x(_) ±SD, n = 6).

Parameters	RTV (control)	XXPI (1 day) + RTV	XXPI (7 days) + RTV
Tmax (h)	2.33 ± 1.03	3.50 ± 0.55*	1.83 ± 0.98
Cmax (μg/ml)	0.23 ± 0.89	0.32 ± 0.14	0.46 ± 0.25
t1/2,_λz_ (h)	3.70 ± 1.54	6.55 ± 1.99*	5.51 ± 1.15*
AUC_0-12_ (μg·h/mL)	0.62 ± 0.13	1.43 ± 0.73	2.40 ± 1.40
AUC_0-∞_ (μg·h/mL)	0.69 ± 0.11	1.63 ± 0.0.88	2.72 ± 1.40*
V_d_ (L)	17.16 ± 8.90	14.46 ± 7.27	7.59 ± 3.77
CL (L/h)	2.71 ± 1.36	1.53 ± 0.61*	0.94 ± 0.40**
MRTINF (h)	5.28 ± 1.93	7.06 ± 1.70	6.86 ± 1.49

**p< 0.01, *p< 0.05, significantly different compared with control group.

### Effects of XYPI on the Expression of Cyp3a1 Protein in Rat Liver

**FIGURE 3:**
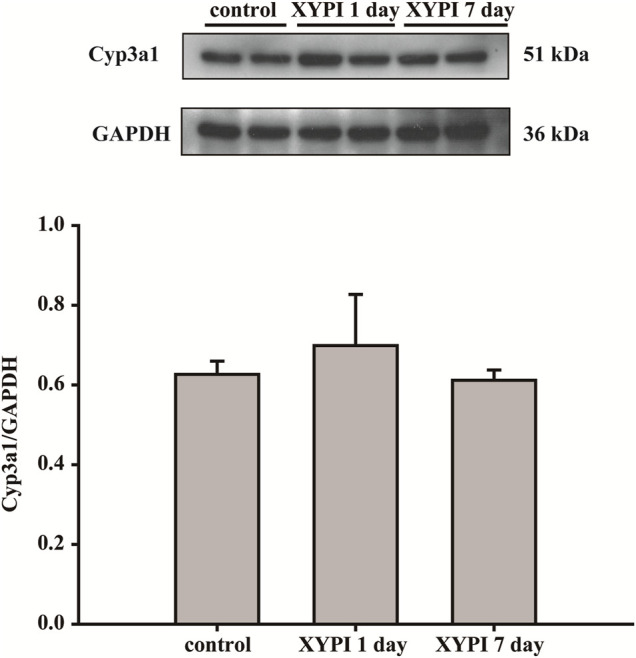
Effects of XYPI on Cyp3a1 protein expression in rat liver.

Our previous study showed that XYPI could inhibit the activity of CYP3A4 in human liver microsome. To assess whether XYPI could affect the Cyp3a1 expression at the protein level in rat liver, western blot assays were performed. As presented in Figure 3, comparing with 1 day, the expression of Cyp3a1 protein was slight decreased after 7 days treatment. However, the Cyp3a1 protein expression after administration of XYPI had no significant difference from control by statistics disposal.

### Inhibitory Effect on CYP3A4

**FIGURE 4:**
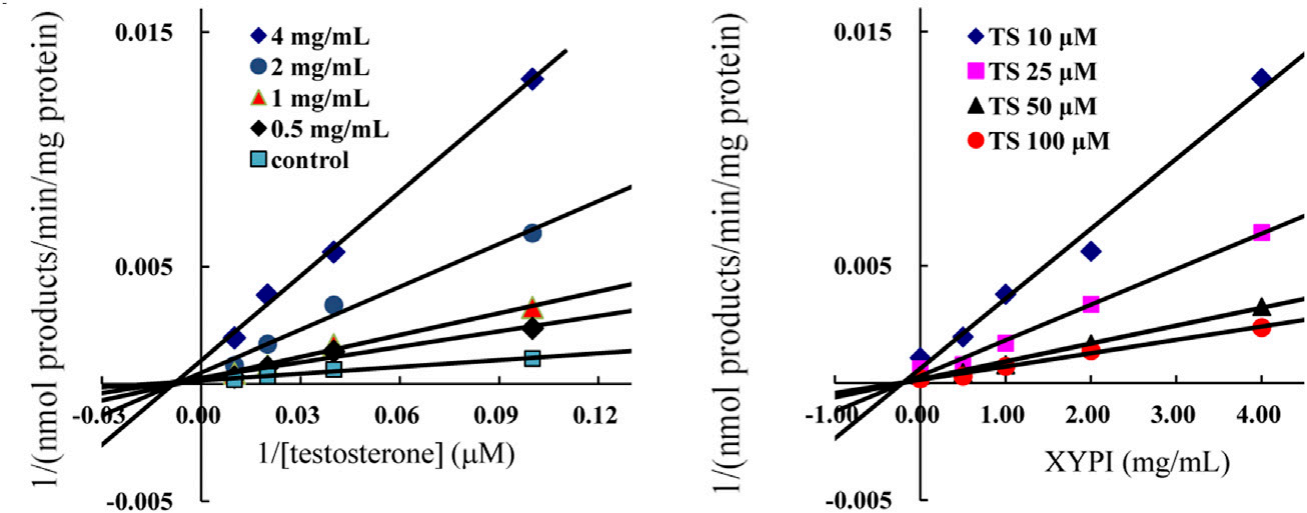
Representative Lineweaver-Burk plots **(A)** and Dixon plots **(B)** of XYPI on CYP3A4-mediated testosterone 6β-hydroxylation metabolism. Each point represents mean ± SD (n = 3).

For further investigating the inhibition model and inhibition constant *Ki* for the XYPI-induced inhibition of CYP3A4, testosterone was used as a probe and 6β-hydroxytestosterone formation was analyzed in liver microsomal reaction systems. The Lineweaver-Burk plots and Dixon plots showed that XYPI noncompetitively inhibited the CYP3A4-catalyzed conversion of testosterone to 6β-hydroxytestosterone with an apparent *Ki* value of 0.54 mg/ml (Figure 4). Collectively, our results confirm that XYPI can inhibit the activity of CYP3A4 isoenzyme and cause potential herb-drug interactions *in vitro* and *in vivo*. The results of our study are consistent with those of a previous study, which indicated that *A. paniculata* extract induced a strong inhibition of CYP3A4 (Pekthong et al., 2008).

**FIGURE 5:**
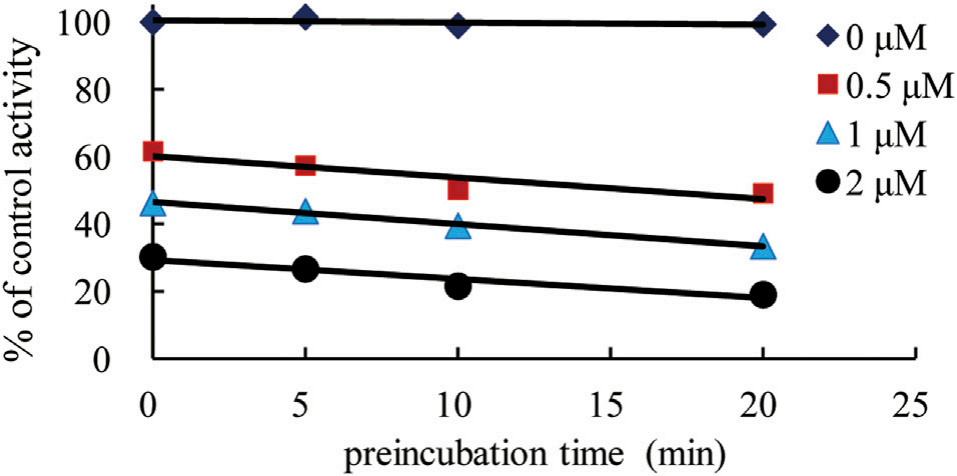
Time-dependent inhibition of CYP3A4 by XYPI.

To further characterize the time-dependent inhibition of XYPI against human CYP3A4, the inactivation of XYPI against CYP3A in HLMs were examined with preincubation times of 0, 5, 10, and 20 min. As shown in Figure 5, the residual enzymatic activities of CYP3A4 decreased gradually with the prolongation of preincubation time, a long preincubation time resulted in much more potent inhibitory effects of XYPI against CYP3A4. The result indicates that XYPI time-dependently inhibited the catalytic activities of CYP3A4.

## Discussion

TCM has been used in clinical practice in China for thousands of years for the prevention and treatment of various diseases including serious epidemic diseases and chronic diseases, and it is still used worldwide. During the outbreak of the COVID-19 epidemic, TCMs were included in the Chinese guidelines for the diagnosis and treatment of COVID-19. Thousands of patients have received treatment with TCMs and have achieved remarkable therapeutic effects (Gao, 2020; Zhuang et al., 2020). XYPI is one of the top 10 recommended TCMs for COVID-19 treatment (Zhuang et al., 2020). It is used for the syndrome of internal exuberance of heat toxin in critical cases of COVID-19 in the progressive stage. LPV/RTV, the current first-line antiretroviral drugs for HIV treatment, presented an antiviral effect against respiratory syndrome coronavirus two and have shown efficacy in COVID-19 patients (Cao et al., 2020); both are metabolized by CYP3A4 (Choy et al., 2020; Nutho et al., 2020). Furthermore, RTV, an inhibitor of CYP3A4, is used at a low dose for markedly increasing plasma LPV concentrations in the compound preparation.

For overcoming the COVID-19 epidemic more quickly and effectively, drug combinations are recommended in the clinic, including drug-drug and/or herb-drug combinations (Deng et al., 2020; Zhang et al., 2021b). However, the combination of drugs may trigger drug-drug/herb-drug interactions, resulting in adverse drug reactions in clinical settings. For example, the combination of Qingfei Paidu decoction with LPV causes HDIs, which may result in adverse drug reactions in patients with COVID-19 through inhibition of CYP3A (Zhang et al., 2021a).

CYP3A4 accounts for approximately 30% of all hepatic P450, and it is responsible for the metabolism of approximately 50% of all currently known therapeutic drugs, which include several drugs being used against the novel coronavirus such as LPV, RTV, chloroquine, and hydroxychloroquine (Deb and Arrighi, 2021), as well as arbidol (Deng et al., 2013). Inhibition of CYP3A4 activity may result in untoward adverse drug interactions in clinical settings because the isoenzyme inhibition may lead to increased plasma levels of a concomitantly administered drug and drug-induced toxicity. On the basis of our previous study, we further explored whether XYPI could inhibit CYP3A4 activity *in vivo* and investigated the XYPI-induced inhibition of CYP3A4 in an *in vitro* model.

According to the theory of TCM and related guidelines, certain TCMs do require long-term and repeat doses in clinical practice. In this study, to simulate clinical anti-coronavirus therapy, rats were administrated LPV/RTV once for 2 h and seven consecutive days after XYPI administration. The group subjected to a single administration of XYPI showed no significant change in the pharmacokinetic parameters of LPV compared with the control group, which indicated that single pretreatment of XYPI might have limited effected on the pharmacokinetics of LPV. After administration of XYPI doses for seven consecutive days, the pharmacokinetic parameters of LPV was only slightly changed, except for the half-life had a significant prolongation. However, the AUC_0-∞_, t_1/2_ and CL of RTV was significantly changed after multiple dosing.

Although the changes of Cmax were not statistically significant, the plasma concentrations of LPV and RTV were increased from 9.94 μg/ml to 11.88 μg/ml (*p* > 0.05) and 0.23 μg/ml to 0.46 μg/ml, respectively. We observed that RTV exposure increased more than 2 times in the 1-day group and significantly about 4 times in the 7-days group, compared with that in the control group. Surprisingly, the increase in LPV exposure was not consistent with the increase in RTV exposure. There was no significant increase in exposure to LPV in the 1-day group and only 0.5 times increase in exposure in the 7-days group (*p* > 0.05).

In order to further explore whether the effect of XYPI on the pharmacokinetics of LPV and RTV is related to the inhibition of CYP3A protein level, we assessed the expression of Cyp3a1 in rats. However, the results showed that the expression of CYP3A protein was not significantly changed in the administration groups compared with the control group. Therefore, in addition to the influence of protein expression changes, we speculated that there may be several reasons for influencing pharmacokinetic changes. First, it indicated that RTV inhibits the metabolism of LPV with an IC_50_ value of 50 ng/ml in human liver microsomes; 50% of LPV was inhibited by RTV at an RTV plasma concentration of 360 ng/ml in HIV-infected patients (Moltó et al., 2008). In our current study, in the 1-day group and 7-days group administered XYPI, the C_max_ of RTV was 0.32 μg/ml (lower than 0.36 μg/ml) and 0.46 μg/ml, respectively. In general, considerably higher blood concentrations of RTV are needed for inhibiting the metabolism of LPV in rats, which indicates that the increase in RTV concentration was too low to affect LPV metabolism in this study. Second, several CYP3A4 substrates overlap with P-gp substrates, LPV and RTV are both substrates of CYP3A4 and P-gp, the concentration of LPV is higher than that of RTV, and LPV out-competes RTV for binding to the P-gp protein in the LPV/RTV combination therapy, which accelerates the efflux of LPV (du Plooy et al., 2011). Additionally, LPV has been reported to be effluxed by both P-gp and MRP2, resulting in its poor oral bioavailability (Agarwal et al., 2007). The interaction between CYP3A and P-gp is highly complex; they may act in synergy. In addition, several factors may affect the blood concentration of LPV including weight, food ingestion, and the blood C reactive protein levels (Stöhr et al., 2010; Alvarez et al., 2021).

Lineweaver-Burkplots and Dixon plots were constructed using the inhibition data for the XYPI-mediated testosterone 6β-hydroxylation activity for clarifying the mode of action *in vitro*. The result showed that XYPI noncompetitively inhibited CYP3A4 with a *Ki* value of 0.54 mg/ml, which is consistent with the results of previous studies (Pekthong et al., 2008; Pekthong et al., 2009; Qiu et al., 2012). *A. paniculata* extract has been reported to significantly decrease CYP3A4 expression and activity in human hepatocytes (Pekthong et al., 2009). Andrographolide, the major constituent of *A. paniculata*, reduced the metabolic activity of intestinal CYP3A4 by significantly down-regulating the mRNA and protein levels of CYP3A4 (Qiu et al., 2012). It reported that some marketed antiviral herbal medicine can inhibit P450 in a time-dependent manner (Zhang et al., 2021b). So, we next analysed whether the herbal medicine is involved in time-dependently inhibition on human CYP3A4, and then different preincubation periods were investigated in HLMs in the presence of XYPI. As expected, a long preincubation time resulted in much more potent inhibitory effects of XYPI against CYP3A4. This result in further do support that continuous administration of XYPI has more effect on the pharmacokinetic of LPV and RTV. Collectively, these results indicate that XYPI can affect the metabolism of LPV/RTV and cause a drug interaction by inhibiting the activity of CYP3A4.

LPV/RTV has been reported to exhibit adverse drug reactions including diarrhea, nausea, weakness or fatigue, headache, vomiting, abdominal pain, rash, hyperlipemia, abnormal liver function and cardiac adverse effects (Cvetkovic and Goa, 2003; Gérard et al., 2020). In addition, fatal pancreatitis and increased bleeding in hemophiliacs have been reported. Notably, almost a quarter of patients in clinical studies presented a serious or fatal laboratory test abnormality. The herb-drug interaction between XYPI and LPV/RTV may result in dose-related adverse drug reactions and become more serious after continuous co-administration for several days.

CYP3A4 has been reported to be the most impacted isoform in inflammation-related P450 down-regulation, which might lead to lower metabolism, prolonged half-life, and increased plasma concentration of CYP3A4 substrate drugs used for COVID-19 treatment (Deb and Arrighi, 2021). Inhibition of the metabolic enzyme system, which results in disruption of drug metabolism, might lead to organ damage and higher mortality rate in patients severely ill from COVID-19 (Deb and Arrighi, 2021). A recent study has shown that drugs metabolized by P450 enzymes exhibited nearly a four times higher likelihood of causing drug-induced liver injury (Sarges et al., 2016). Clinical research indicates that the use of LPV/RTV leads to a 4-fold increased odds of liver injury in COVID-19 Patients (Cai Q. et al., 2020). Thus, pharmacists and physicians should pay considerable attention toward CYP3A4, because most drugs used for COVID-19 treatment in the clinic are metabolized by this isoform; drug-drug/herb-drug interactions; and, above all, drug-disease interactions caused by drug combinations used in the treatment of COVID-19.

Potential herb-drug interaction was found significant for RTV, which in this combination is used in a subtherapeutic dose for “boosting” the LPV concentration by inhibition of CYP3A4; and even though the AUC for RTV is significantly increased, this does not signally affect the AUC for LPV and so the clinical significance of this interaction may be limited. However, extreme caution should be taken regarding when LPV/RTV is used combination with XYPI in clinical practice.

When it comes to potential clinical drug-interaction between LPV/RTV and XYPI, there are a number of issues to consider. First, it is important to note that animal experiments and/or *in vitro* inhibition observed in this study may or may not translate into measurable in clinical practice. Second, it has been shown that plasma concentrations of LPV in COVID-19-infected patients (at the dose regimen of 400/100 mg) was increased compared with HIV-infected patients (Lê et al., 2020). The main reason is associated with infection of SARS-CoV-2 elicitation a high production of cytokines including IL-1, TNF-α and IFN, which lead to down-regulate CYP3A4 and decrease elimination rate of LPV/RTV (Aitken and Morgan, 2007). Under this condition, combined administration with XYPI may further increase plasma concentration of LPV/RTV, lead to the occurrence of hepatotoxicity. In addition, previous study showed a high incidence of adverse events when a higher than standard dose of the LPV/RTV was combined with rifampin (Nijland et al., 2008), which is used as a recommended positive P450 enzymes and P-gp inducer. It suggests that affecting the P450 enzyme and/or may also increase the hepatotoxicity of LPV/RTV. Actually, except for CYP3A4, CYP2D6 and CYP1A2 also contribute to LPV metabolism. And 2,6-dimethylphenol (DMP) is a metabolite of LPV mediate by CYP1A2, which can be further metabolized to form quinine and/or quinone methide intermediates and resulted in drug-induced toxicity (Bolton et al., 2000; Evans et al., 2004). Reduction in formation of DMP *via* inhibition of CYP1A2 by *A. paniculata* extract preparation may be a potentially beneficial aspect of this interaction (Chien et al., 2010). In this case, XYPI might protect patient from this side effect of LPV/RTV therapy.

## Conclusion

The present study confirms potential herb-drug interactions between XYPI and LPV/RTV through *in vitro* and *in vivo* studies for the first time. The results clearly demonstrated that XYPI affected the pharmacokinetics of LPV/RTV through strong noncompetitive inhibition of CYP3A4 in a time-dependent manner. Extreme caution needs to be taken when XYPI is co-administrated with LPV/RTV for COVID-19 treatment. Patients taking herb medicines should inform their physicians before being prescribed therapies with Western drugs to avoid the potential risks of drug interactions. In addition, plasma drug levels need to be measured for drugs with a narrow therapeutic index for minimizing drug-related toxicity and optimizing the pharmacotherapy of COVID-19 in the clinical setting.

## Data Availability

The original contributions presented in the study are included in the article/Supplementary Material, further inquiries can be directed to the corresponding author.
