# Supplementary material for: Herb-Drug Interaction Between Xiyanping Injection and Lopinavir/Ritonavir, Two Agents Used in COVID-19 Pharmacotherapy
Source: Front Pharmacol. 2021 Nov 11;12:773126. doi: 10.3389/fphar.2021.773126 (PMC8660086; doi:10.3389/fphar.2021.773126)
Supplement: Supplementary file 1 [file Table1.DOCX]

| Table S.1 Precision and accuracy of LC-MS/MS method for the determination of LPV and RTV in rat plasma | | | | | | | |
| --- | --- | --- | --- | --- | --- | --- | --- |
| Analytes | Concentration  (ng/mL) | Intra batch (n = 6) | | | Inter batch (n=6) | | |
|  |  | Determined  (ng/mL) | RE (%) | RSD  (%) | Determined (ng/mL) | RE (%) | RSD (%) |
| LPV | 90 | 99.57 | 10.63 | 2.31 | 92.41 | 2.68 | 9.38 |
|  | 900 | 896.33 | 0.41 | 4.96 | 848.6 | 5.71 | 10.66 |
|  | 7500 | 7061.67 | 5.84 | 6.54 | 7493.33 | 0.09 | 8.43 |
| RTV | 15 | 13.75 | 8.33 | 4.55 | 14.55 | 3.01 | 5.64 |
|  | 150 | 152.00 | 1.33 | 3.69 | 156.29 | 4.19 | 6.36 |
|  | 1500 | 1413.33 | 5.78 | 2.68 | 1405.58 | 6.29 | 7.20 |
